# Characterization of Free, Conjugated, and Bound Phenolic Acids in Seven Commonly Consumed Vegetables

**DOI:** 10.3390/molecules22111878

**Published:** 2017-11-01

**Authors:** Yuan Gao, Shuai Ma, Meng Wang, Xiao-Yuan Feng

**Affiliations:** 1 Beijing Research Center for Agricultural Standards and Testing, No. 9 Middle Road of Shuguanghuayuan, Haidian Dist., Beijing 100097, China; gaoy@brcast.org.cn (Y.G.); mas@brcast.org.cn (S.M.); wangm@brcast.org.cn (M.W.); 2 Risk Assessment Laboratory for Agro-products (Beijing), Ministry of Agriculture, No. 9 Middle Road of Shuguanghuayuan, Haidian Dist., Beijing 100097, China

**Keywords:** phenolic acid, vegetables, region, characteristic components, PCA

## Abstract

Phenolic acids are thought to be beneficial for human health and responsible for vegetables’ health-promoting properties. Free, conjugated, and bound phenolic acids of seven commonly consumed vegetables, including kidney bean, cow pea, snow pea, hyacinth bean, green soy bean, soybean sprouts and daylily, from the regions of Beijing, Hangzhou, and Guangzhou, were identified and quantified by ultra-performance liquid chromatography-tandem mass spectrometry (UPLC-MS/MS). Three vegetables, namely green soy bean, soybean sprouts, and daylily (*Hemerocallis fulva* L.), from the Beijing region contained higher concentrations of total phenolic acids than those from the Hangzhou and Guangzhou regions. The results indicated that the phenolic acid content in the seven vegetables appeared to be species-dependent. The highest content of phenolic acids was found in daylily, followed by green soy bean, while the least amounts were identified in kidney bean and hyacinth bean. Typically, phenolic acids are predominantly found in conjugated forms. Principle component analysis (PCA) revealed some key compounds that differentiated the seven vegetables. Green soy bean, compared to the other six vegetables, was characterized by higher levels of syringic acid, ferulic acid, vanillic acid, and sinapic acid. Other compounds, particularly *p*-coumaric acid, neochlorogenic acid, and caffeic acid, exhibited significantly higher concentrations in daylily. In addition, *p*-coumaric acid was the characteristic substance in cow pea. Results from this study can contribute to the development of vegetables with specific phytochemicals and health benefits.

## 1. Introduction

As basic consumable food products in people’s daily lives, vegetables constitute an increasing amount of the diet, especially in China, reaching up to 33.7% of the total dietary consumption [1]. Epidemiological studies have provided evidence to prove that the frequent consumption of fruits and vegetables is associated with a reduced risk for some chronic and cardiovascular diseases [2]. In particular, phenolic compounds are one of the most important natural antioxidants responsible for these health promoting properties due to their bioactivities [3,4]. Phenolics, as a product of secondary metabolism, are considered to deliver health benefits by free radical scavenging, antioxidant, anticarcinogenic, anti-inflammatory, or antimicrobial effects [5,6,7]. Among the variety of phenolic compounds, phenolic acids have attracted considerable interest. Many studies showed that phenolic acid significantly contributes to plant development, induced resistance, sensory qualities, and flavors [8,9]. The main dietary sources of phenolic acids are fruits, vegetables, cereals, and beans [3,10]. However, levels of phenolic acids found in these sources maybe affected by geographical region, variety, cultivation, climate, and extraction process [11,12].

Some studies in the literature have focused on the extraction and analysis of phenolic acids from vegetables, including potatoes [11], tomatoes [13], eggplants [14], carrots [15], Chinese cabbage [16,17], bitter melons [18], and broccoli [19]. Chlorogenic acid was the most studied phenolic and showed relatively high levels in eggplants and potatoes. Ferulic acid, caffeic acid, sinapic acid, *p*-hydroxybenzonic acid, and *p*-coumaric acids have also been studied. Some literature about phenolic acids in dry beans also exists. Darmadi-Blackberry and colleagues reported that consumption of legumes, including dry beans, was the most critical factor compared to other food in determining longevity [20]. According to epidemiological evidence, the protective effect of reducing chronic diseases through the intake of more vegetables are attributed, in part, to the occurrence of different antioxidant components, and mainly to phenolic compounds [4,21]. Bean extracts from white kidney beans and round purple beans have been proven to be rich in phenolics and exhibit both antioxidant and anti-inflammatory activities [21]. However, published data on the contents of phenolic acids are limited for fresh beans, such as kidney bean (*Phaseolus vulgaris* L.), cow pea (*Vigna unguiculata*), and hyacinth bean (*Lablab purpureus*). Most of the literature focused on the determination of total polyphenols by Folin-Ciocalteu colorimetry in these above vegetables [22]. Additionally, daylily (*Hemerocallis fulva* L.) and soybean sprouts, as traditional Chinese food, are consumed widely and deeply loved by people for their high nutrition value. Studies also showed that the extracts from daylily had strong antioxidant activity and scavenging effects on free radicals and nitric oxide [23]. Nonetheless, comprehensive food composition data for specific phenolic acid content in these vegetables are still lacking.

Phenolic acids in plants may exist in free, soluble conjugated (esterified), and insoluble-bound forms [24,25,26]. However, soluble conjugated phenolic acids have not received as much attention as the free forms. Studies demonstrated that phenolic acid conjugates are recognized antioxidants with anti-inflammatory properties both in vitro and in vivo [27,28,29,30]. Conjugated and bound phenolics may also play an essential role in delivering antioxidants to the colon upon their release by bacterial microbiota [31]. These two forms of phenolic acids, considered to be bound to oligosaccharides, peptides or polysaccharides, can be released after acidic or alkaline hydrolysis. Therefore, there is demand for a comprehensive analysis of all forms of free, conjugated, and bound phenolics.

Most of the studies so far have only been concerned with total phenols or have only considered a few extractable free phenolics present in fruits and vegetables. The evolution and distribution of three types of phenolic acids in these commonly consumed vegetables have not been well investigated. As such, demand exists for a comprehensive analysis of phenolic acid. In this study, we aimed to evaluate the phenolic acids, including free, conjugated, and bound forms in seven vegetables, including kidney bean, yardlong cow pea, snow pea, hyacinth bean, green soy bean, soybean sprouts and daylily, from three regions in China, Beijing, Hangzhou, and Guangzhou, using ultra-performance liquid chromatography-tandem mass spectrometry (UPLC-MS/MS). The distribution, existing forms, and characteristic substances of these vegetables were investigated. The results of this study can provide further insights into the health-promoting compounds of vegetables, and offer a more in-depth bioactivities index for further research.

## 2. Results and Discussion

Phenolic acids in plants may exist in three types: free, soluble conjugated, and insoluble bound types. Free-form phenolics are directly released and easily detected, while the conjugated and bound forms need to be alkaline hydrolysed before being perceived during processing or storage. Generally, phenolic acids are divided into hydroxybenzoic and hydroycinnamic acids according to their structural features.

### 2.1. Free Phenolic Acid

The free, conjugated, and bound forms of phenolic acids in the seven vegetables are shown in Table 1, Table 2 and Table 3. Our results indicate that among the three groups, the free-form phenolic acids accounted for the lowest proportional concentration in these vegetables. With respect to individual phenolic acid, six free phenolics were identified and quantified, including three hydroxybenzoic acids, vanillic acid, *p*-hydroxybenzoic acid and protocatechuic acid, and three hydroxycinnamic acids, neochlorogenic acid, chlorogenic acid and *p*-coumaric acid (Table 1). Both the region and species had influences on the free phenolic acids content. Generally, the subtotal free phenolic acids of green soy bean, soybean sprouts, and daylily showed markedly higher concentrations in the Beijing region compared to the Hangzhou and Guangzhou regions. Apart from chlorogenic acid and vanillic acid, the other phenolics all had higher concentrations in daylily from the Beijing region than from the other two regions. So far, no studies exist on the regional variations in phenolic compounds in the above vegetables.

Regarding the seven different vegetables, our results show that the types and content of free phenolic acids vary considerably. Except for daylily, only one or two phenolic acids were detected in the other six vegetables. For instance, two free-form phenolic acids, chlorogenic acid and *p*-coumaric acid, were detected in kidney bean and cow pea, and the levels were lower than the values in green beans obtained by Mazzeo et al. [32]. This could be attributed to various factors, such as cultivar genotype, geographical region, climate, and assay procedure [12,33,34]. Meanwhile, *p*-coumaric acid was detected in minute amounts in snow pea (0.09–0.22 mg/kg fresh weight (FW)). According to the published data, only the determination of total polyphenols by Folin-Ciocalteu colorimetry in cow pea and snow pea exists [22]. For the free-form phenolics, *p*-hydroxybenzoic acid was the only phenolic acid detected in green soy bean and soybean sprouts. Free phenolic acid was undetectable in hyacinth bean from any of the three regions. To our knowledge, no information was available on the phenolics in hyacinth bean and green soy bean. The highest concentration of free phenolic acids was observed in daylily, and a total of seven free form phenolics were detected. Among these compounds, neochlorogenic acid was the most abundant acid in daylily, ranging from 84.04 mg/kg FW in Hangzhou region to 103.10 mg/kg FW in the Beijing region, whereas *p*-coumaric acid showed the lowest amount (4.03–5.11 mg/kg FW). A study showed that the water extract from daylily expressed relatively high antioxidant activity and an inhibitory effect on nitric oxide production [23]. The large amount of phenolic acids in daylily detected in our results also suggests the strong antioxidant capacities. 

So far, only a few reports have studied the concentration of certain phenolic acids in these vegetables. The study of Silva et al. found four phenolic acids in soybean sprouts, including caffeic acid, neochlorogenic acid, *p*-coumaric acid, and ferulic acid, which does not align with our results. The reason for the difference may be that, in addition to cultivar genotype, the extraction process also played an important role on the levels of phenolic acids found. In their study, freeze-dried sprouts were boiled for 15 min to mimic the way how the sprouts are usually prepared for human consumption [35]. Hence, in the process, the conjugated or bound phenolic acid may be released by thermal hydrolysis. In our results, several conjugated and bound phenolic acids were also detected in soybean sprouts. 

### 2.2. Conjugated Phenolic Acid

The conjugated phenolic acids in the seven vegetables were assessed (Table 2). Six hydroxybenzoic acids, including gallic acid, 2,3,4-trihydroxybenzoic acid, vanillic acid, *p*-hydroxybenzoic acid, syringic acid, and protocatechuic acid and four hydroxycinnamic acids, including ferulic acid, caffeic acid, *p*-coumaric acid, and sinapic acid, were detected. The seven vegetables in the three regions presented somewhat different phenolic amounts. Three vegetables, namely green soy bean, soybean sprouts, and daylily, from the Beijing region all showed relatively higher levels of total conjugated phenolic acids compared to those from the Hangzhou and Guangzhou regions. The difference in the amounts of total phenolic acids may be due to regional climate, and growing and storage conditions [36].

The region factor showed less of an effect than species difference on phenolic acid metabolism. Large differences were found in the levels of conjugated phenolic acids among the seven different vegetables. Our results indicate that the conjugated phenolic acids accounted for the majority of the phenolic acids in all of the seven vegetables and accounted for the largest proportion of the three forms, which aligns with the results obtained from cranberry beans (*Phaseolus vulgaris* L.) [10]. For the conjugated fraction, the three phenolic acids present in all the vegetables were gallic acid, ferulic acid, and *p*-coumaric acid. Additionally, *p*-coumaric acid was the predominant conjugated phenolic acid in most of the samples in the current study, except for in hyacinth and green soy beans. Hyacinth bean contains more protocatechuic acid than any other phenolic acid, and syringic acid showed the highest level in green soy bean. However, apart from green soy bean, syringic acids were only detected in daylily in extremely low levels. 

Ferulic acid is considered to have a structural role in cross-linking wall polymers and some research suggests that ferulic acid is the most predominant phenolic acid in dry edible beans [33]. Our results showed that ferulic acid was the second highest compound detected in most of the vegetables and this component was particularly high in green soy bean (32.67–46.61 mg/kg FW) and daylily (37.86–40.10 mg/kg FW), but showed relatively low levels in kidney bean (1.17–3.0 mg/kg FW). Additionally, vanillic acid and *p*-hydroxybenzoic acid were only detected in green soy bean, soybean sprouts, and daylily. 2,3,4-trihydroxybenzoic acid was identified in quantifiable amount only in kidney bean, soybean sprouts, and daylily. 

Daylily and green soy bean, as typical Chinese food, contain high levels of phenolics. According to our results, the highest amount of total conjugated phenolic acids was found in daylily (300.58–313.63 mg/kg FW), followed by green soy bean (192.58–238.79 mg/kg FW). *p*-coumaric acid accounted for about 45% of the total conjugated forms in daylily, with intermediate levels of caffeic acid (25%) and ferulic acid (15%). Moreover, conjugated *p*-coumaric acid had the highest level in daylily (134.66–149.61 mg/kg FW) compared to the other six vegetables. Notably, for snow pea and cow pea, *p*-coumaric acid accounted for the largest proportion of the conjugated fraction, with nearly 70% and 90% of the total phenolics, respectively. In addition, insignificant amounts of conjugated caffeic acid were extracted in the other vegetables, except for daylily (69.54–73.42 mg/kg FW). Quantitatively, syringic acid, ranging from 87.45 mg/kg FW in Hangzhou region to 102.46 mg/kg FW in the Beijing region, accounted for more than 40% of the total conjugated fractions in green soy bean. Except for syringic acid, ferulic acid (32.67–46.61 mg/kg FW), vanillic acid (26.88–41.59 mg/kg FW), and sinapic acid (32.93–42.01 mg/kg FW) were the other three conjugated fractions with higher concentrations detected in green soy beans, accounted for about 20% of the total. Conjugated phenolic acids are soluble components extractable by methanol aqueous solution, but considered to be bound to soluble oligosaccharides and peptides through ester bonds or ether linkage, which can be released after hydrolysis [31,37]. Phenolic acids were predominantly found in conjugated forms [10]. The same results were obtained in our study. However, a study stated that the majority of phenolic acids were extracted from the alkaline hydrolyzed fraction, and further sequential acid hydrolysis of the same extract did not yield any additional amounts of phenolic acid [33], which means most conjugated phenolic acids were more likely to be released by alkaline hydrolysis rather than acid hydrolysis [10,30]. Only three phenolic acids, namely the conjugated forms of *p*-coumaric acid, ferulic acid, and sinapic acids, were previously reported in dry beans [10], and no information was available for the vegetables used in our study. This is a significant finding, since the analytical methods commonly used to determine the amount of phenolic acids for the above vegetables in previous studies were direct extraction and injection without hydrolysis, which only provide the free forms. Hence, this could lead to significant underestimation of the total extractable phenolic content of a particular food [38,39]. Conjugated and bound phenolics may play an essential role in delivering antioxidants to the colon, upon their release by bacterial microbiota [31,40]. Our research on conjugated phenolic acids can help to better understand the antioxidant effects and nutritional value of these vegetables.

### 2.3. Bound Phenolic Acid

Bound phenolic compounds are esterified to cell wall polysaccharides, but can also be covalently linked to lignin monomers with an ether linkage [10]. These phenolic acids are the insoluble phenolics remaining in the residue following the initial extraction with 80% MeOH. Table 3 show the bound phenolic acids in the seven vegetables. Bound phenolics, released upon alkaline hydrolysis, contained a total of eight substances, including four hydroxybenzoic acids (vanillic acid, *p*-hydroxybenzoic acid, syringic acid, and protocatechuic acid) and four hydroxycinnamic acid (ferulic acid, caffeic acid, *p*-coumaric acid, and sinapic acid). The accumulation of bound phenolics varied between regions and species. Similarly, the levels of total bound phenolic concentrations detected in green soy bean, soybean sprouts, and daylily were significantly higher in the Beijing region than in the other two regions. 

*p*-Coumaric acid was the only bound phenolic found in all the seven vegetables, but large differences in the levels were found. The bound form of vanillic acid, *p*-hydroxybenzoic acid, and syringic acid were detected in green soy bean, soybean sprouts, and daylily. Sinapic acid was identified in measurable amounts only in snow pea and green soy bean. Caffeic acid was also released as a bound phenolic compound, but was only detected in hyacinth bean and daylily in relatively small amounts. Quantitatively, bound-form phenolic acids were characterized in small amounts in kidney bean, hyacinth bean, snow pea, and cow pea. In fact, *p*-coumaric acid was the only phenolic acid in the bound form detected in kidney bean and cow pea. The highest subtotal levels of bound phenolics were found in green soy bean, followed by daylily. The subtotals of bound phenolics in green soy bean represented a significant portion (about 30%) of the total phenolic index, including the subtotals of the free and conjugated fractions. Syringic acid was the predominant bound fraction in green soy bean (33.40–45.10 mg/kg FW), which was the same as the conjugated forms. Ferulic acid, vanillic acid, and sinapic acid were also found in significant amounts in green soy bean. In addition, syringic acid was found to be the most abundant acid in soybean sprouts (10.22–17.63 mg/kg FW), while daylily contained the highest concentration of *p*-coumaric acid (16.31–22.43 mg/kg FW). 

To date, no literature has reported on bound-form phenolic acids in these vegetables. Previous studies only provided a partial characterization of phenolics. To the best of our knowledge, this is the first time all possible phenolic acids in these vegetables were characterized. Finding a significant amount of conjugated and bound phenolic acids, as detected and quantified in this study, provides systematic estimation of biological activities, including beneficial health effects.

### 2.4. Principal Component Analysis

**Figure 1:**
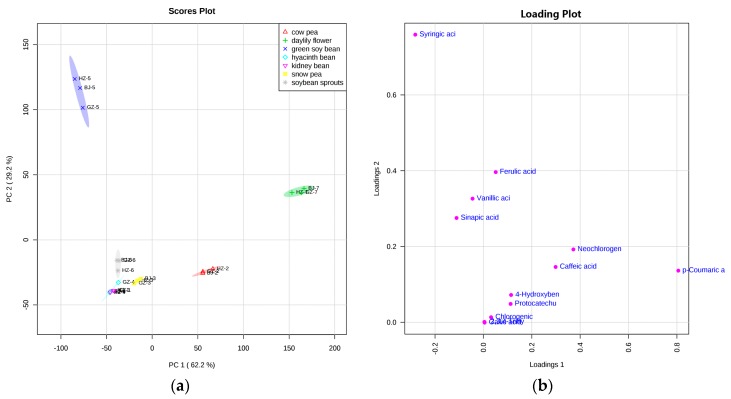
Principal component scatter plot and corresponding loading plot of phenolic acids: (**a**) score scatter plot and (**b**) loading plot. Each data point was the average of three replications, *n =* 3. BJ: Beijing; HZ: Hangzhou; GZ: Guangzhou. The numbers (1–7) following the letter represented region represented kidney bean, cow pea, snow pea, hyacinth bean, green soy bean, soybean sprouts, and daylily, respectively.

To provide an overview of the effects of region and variety on the phenolic acid composition and to further identify the discriminant components, principal component analysis (PCA) was applied using all the detected phenolics as variables. All the seven vegetables, taking into account the three regions, were used for the PCA. The PCA score scatter plot of all samples is shown in Figure 1a. The series of numbers (1–7) following the letter represented region (BJ, HZ, and GZ) represented kidney bean, cow pea, snow pea, hyacinth bean, green soy bean, soybean sprouts, and daylily, respectively. And the corresponding loading plot is shown in Figure 1b, establishing the relative importance of the variables. Based on all the detected phenolic acids, using the total concentrations of their respective free, conjugated and, bound types, the first two principal components (PCs) accounted for about 91.4% of the total variance. As shown in the loading plot (Figure 1a), the first component (PC1) explained approximately 62.2%, and the samples were almost distributed on the negative axis of PC1, except for cow pea and daylily from all three regions. PC2 accounted for 29.2% of the total variance. Except for two vegetables (green soy bean and daylily, from all three regions), all the other five vegetables were distributed on the negative axis of PC2.

Our results show that the seven vegetables were separated from each other, and the semi-transparent fields meant 95% confidence intervals. In combination with the corresponding loading plot, green soy bean, compared with the other six vegetables, was characterized by high levels of syringic acid. Simultaneously, ferulic acid, vanillic acid, and sinapic acid also had a bias toward green soy bean, suggesting the high levels detected in green soy bean. As shown in Figure 1, *p*-coumaric acid, neochlorogenic acid, and caffeic acid were the characteristic substances in daylily; that is, these compounds generally had higher levels in daylily in comparison with the other vegetables. Other compounds, including *p*-hydroxybenzoic acid, protocatechuic acid, chlorogenic acid, gallic acid, and 2,3,4-trihydroxybenzoic acid, were also found to be closer to the points representing daylily from all three regions (Figure S1). According to the distance between the points representing these four vegetables, namely soy bean sprouts, hyacinth bean, kidney bean, and snow pea, and phenolic acids attributers (Figure S1), these four vegetables contain relatively lower levels of phenolics. In addition, *p*-coumaric acid was the characteristic compound in cow pea, which is a critical distinguishing factor between cow pea and other legume vegetables. 

## 3. Materials and Methods 

### 3.1. Sampling and Processing

Vegetables were selected taking into account the same seasonality and volume of consumption in China. Seven vegetables, namely kidney bean (*Phaseolus vulgaris* L.), yardlong cow pea (*Vigna unguiculata* (*Linn.*) *Walp*), snow pea (*Pisum sativum var. macrocarpon* L.), hyacinth bean (*Lablab purpureus* (*Linn.*)), green soy bean (*Glycine max* (L.) *Merr*), soybean sprouts, and daylily flower (*Hemerocallis fulva* L.) were collected from three regions, including Beijing, Hangzhou and Guangzhou. For representative purpose, each kind of vegetable from each region were sampled randomly from five stalls, including three wholesale vegetable markets and two hypermarkets, for a total of 2 kg each, and then mixed together as one sampling point. All the sampling points were divided into three sampling units to create three biological replicates, 2 kg per replicate. Vegetables were placed in a bubble chamber and transported on ice to the laboratory within 24 h. The inedible parts of the raw samples were removed manually with a sharp knife on an ice plate and cut into slices of 8–10 mm in length before being frozen in liquid nitrogen and then stored at −80 °C until analysis. All the samples were analyzed within one month’s time.

### 3.2. Chemicals and Standards

Sodium hydroxide (NaOH) and hydrochloric acid (HCl) (analytical grade) were purchased from Beijing Chemical Reagent Company (Beijing, China). Ascorbic acid (Vc), gallic acid, 3,5-dihydroxybenzoic acid, neochlorogenic acid, protocatechuic acid, 2,3,4-trihydroxybenzoic acid, chlorogenic acid, *p*-hydroxybenzoic acid, gentisic acid, caffeic acid, vanillic acid, syringic acid, pyrocatechuic acid, ellagic acid, *p*-coumaric acid, sinapic acid, ferulic acid, isoferulic acid, *m*-coumaric acid, *o*-coumaric acid, salicylic acid and cinnamic acid were purchased from Sigma-Aldrich (St. Louis, MO, USA). All the standards used for identification and quantification in this study were of HPLC quality. Methanol, formic acid, acetonitrile (MS grade) were received from Thermo Fisher Scientific (Fair Lawn, NJ, USA). Ultrapure water was obtained from a Milli-Q Element water purification system (Millipore, Bedford, MA, USA).

### 3.3. Free Phenolic Acid Extraction

Free phenolic acid extraction followed the method in our previous study with minor modifications [30]. Before analysis, vegetables were grounded to a fine power under liquid nitrogen, and the powder was divided into three subsamples. A subsample (2 g) was mixed with 80% methanol (20 mL) containing 1% ascorbic acid. The resulting mixture was ultrasonicated for 30 min at room temperature and then centrifuged at 10,000 rpm for 10 min. The supernatant was collected and the above extraction was repeated twice more. The combined supernatant were transferred to a 50-mL volumetric flask, diluted with extracting solution to volume, mixed, and then filtered through 0.22 µm PTFE membranes (Pall, Ann Arbor, MI, USA) prior to UPLC-MS/MS analysis.

### 3.4. Conjugated Phenolic Acid Extraction

Conjugated phenolic acid extraction was performed according to Li et al. [41]. The powder (2 g) was mixed with 20 mL of 80% methanol containing 1% ascorbic acid, followed by ultrasonication for 30 min at room temperature. The mixture was centrifuged at 10,000 rpm for 10 min and the extraction was repeated twice. The solid residues were used for the next extraction of bound phenolic acid. The supernatant was combined and evaporated to a volume of less than 10 mL (aqueous phases) at 35 °C using a rotary evaporator. After evaporation, 20 mL of 4 M NaOH was added to the remaining aqueous layer, and the medium in nitrogen blanketing was alkaline hydrolysed by shaking at 40 °C for 2 h. Afterward, the hydrolysate was acidified to pH 2 with 12 M HCl and then 20 mL hexane was added, vibrating for 20 min under ambient temperature. After removing the hexane, the resultant hydrolysate was extracted three times with 20 mL ethyl acetate. In this step, free and conjugated phenolic acids were both extracted with ethyl acetate. Therefore, the levels of conjugated phenolic acids were the difference between the values obtained from this step and the previous step. All the organic phases were pooled and evaporated to dryness at 35 °C. The resultant dry residue was re-dissolved in 10 mL of 50% methanol/ultrapure water (*v*/*v*), and then filtered through a 0.22 µm PTFE membrane filter before further analysis.

### 3.5. Bound Phenolic Acid Extraction

The solid residues remaining after the last step were treated with 20 mL of 4 M NaOH and alkaline hydrolysed by shaking for 2 h at 40 °C under N_2_ as described above. The resultant hydrolysate was acidified to pH 2 with 12 M HCl. Twenty milliliters of hexane were added and shaken for 20 min to remove the oil and other esters. Then, the liberated phenolics were extracted three times with 20 mL ethyl acetate after the hexane was removed. Subsequently, the combined supernatant was evaporated to dryness under vacuum at 35 °C and re-dissolved in 10 mL 50% methanol. Eventually, the released bound phenolic acids were analyzed using the UPLC-MS/MS after filtration through 0.22 µm PTFE membrane filters.

### 3.6. UPLC-MS/MS Analysis

An ACQUITY HSS C18 column (1.8 μm particle size; 2.1 × 150 mm, Waters, Milford, MA, USA) was used for the separation of phenolic acids on a Waters ACQUITY UPLC system interfaced to a triple quadrupole MS (TQ-S, Waters Micromass, Manchester, UK) and an orthogonal Z-spray electrospray ionization (ESI) with Masslynx 4.1 software (Waters, Milford, MA, USA). A gradient consisting of (A) 0.1% formic acid in water (*v*/*v*) and (B) 0.1% formic acid in acetonitrile (*v*/*v*) was applied at a flow rate of 0.3 mL/min. The gradient program was as follows: initial conditions 5% B were held for 30 s, from 5–30% B for 4.5 min, from 30–90% B for 4.5 min, 90% B maintained constantly for 30 s, from 90–5% B for 30 s, and this composition was held for 2.5 min for re-equilibration. The injection volume was 5 μL. The column was maintained at 45 °C and the autosampler was at 10 °C. 

Both positive and negative electrospray ionization (ESI) modes were applied based on the structural properties of phenolic acids. The ESI parameters were as follows: +2.5 kV/−1.0 kV capillary voltage, 150 °C source temperature, 500 °C desolvation temperature; 150 L h^−1^ cone gas flow and 1000 L h^−1^ desolvation gas flow. Detection was performed in multiple reactions monitoring (MRM) mode. Quantification was completed according to the standard curves generated from individual compounds in serial dilutions (1~500 ng mL^−1^).

### 3.7. Statistical Analyses

One-way analysis of variance (ANOVA) was performed using SPSS 20.0 Statistical Package for Windows (SPSS Inc., Chicago, IL, USA) at significance levels of *p* < 0.05. Based on the type and content of phenolic acid, as well as principal component analysis (PCA) of these vegetables from different regions, a scatter plot was created to visualize the difference of phenolics among various vegetables. Each data point was the average of three replications.

## 4. Conclusions

In conclusion, seven commonly consumed vegetables from three regions in China (Beijing, Hangzhou, and Guangzhou), were tested for their phenolic acids contents in free, conjugated, and bound forms. The total amount of phenolic acids in green soy bean, soybean sprouts and daylily showed higher levels in the Beijing region compared to the other two regions. Variety difference had a much stronger influence on the accumulation of phenolic acids. Of all the vegetables, daylily contained the highest levels of free and conjugated phenolic acids, while the highest subtotal amount of bound phenolics was detected in green soy bean. Additionally, conjugated phenolic acids were the main fraction for all seven vegetables and accounted for the largest proportion of the three forms. PCA revealed some key compounds that differentiated the seven vegetables. Most of phenolic acids, especially *p*-coumaric acid, neochlorogenic acid, and caffeic acid, showed significantly higher concentrations in daylily, while green soy bean was characterized by high levels of syringic acid, ferulic acid, vanillic acid, and sinapic acid. Interestingly, *p*-coumaric acid was observed at a high level in cow pea, which is a critical distinguishing factor between cow pea and other legume vegetables. The present study provides comprehensive information on the phenolic acid composition of these vegetables, particularly for conjugated phenolic acids.

## Figures and Tables

**Table 1:** Determination of free phenolic acid content in seven commonly consumed vegetables in China by UPLC-MS/MS (mg/kg FW).

Vegetables	Region	Chlorogenic Acid	Neochlorogenic Acid	*p*-Coumaric Acid	*p*-Hydroxybenzoic Acid	Protocatechuic Acid	Vanillic Acid	Total Free Phenolic Acid
kidney bean	BJ	1.95 ± 0.24 b	n.d.	tr	n.d.	n.d.	n.d.	1.95 ± 0.26 b
	HZ	1.24 ± 0.07 c	n.d.	0.81 ± 0.03 a	n.d.	n.d.	n.d.	2.05 ± 0.11 b
	GZ	2.47 ± 0.27 a	n.d.	0.37 ± 0.02 b	n.d.	n.d.	n.d.	2.84 ± 0.35 a
cow pea	BJ	0.22 ± 0.01 a	n.d.	0.65 ± 0.06 c	n.d.	n.d.	n.d.	0.87 ± 0.06 b
	HZ	0.11 ± 0.00 c	n.d.	1.28 ± 0.17 a	n.d.	n.d.	n.d.	1.39 ± 0.17 a
	GZ	0.15 ± 0.01 b	n.d.	0.79 ± 0.03 b	n.d.	n.d.	n.d.	0.94 ± 0.03 b
snow pea	BJ	n.d.	n.d.	0.09 ± 0.07 c	n.d.	n.d.	n.d.	0.09 ± 0.04 b
	HZ	n.d.	n.d.	0.22 ± 0.01 a	n.d.	n.d.	n.d.	0.22 ± 0.01 a
	GZ	n.d.	n.d.	0.11 ± 0.01 b	n.d.	n.d.	n.d.	0.11 ± 0.01 b
hyacinth bean	BJ	n.d.	n.d.	n.d.	n.d.	n.d.	n.d.	n.d.
	HZ	n.d.	n.d.	n.d.	n.d.	n.d.	n.d.	n.d.
	GZ	n.d.	n.d.	n.d.	n.d.	n.d.	n.d.	n.d.
green soy bean	BJ	n.d.	n.d.	n.d.	0.25 ± 0.01 a	n.d.	n.d.	0.25 ± 0.01 a
	HZ	n.d.	n.d.	n.d.	0.13 ± 0.01 b	n.d.	n.d.	0.13 ± 0.01 b
	GZ	n.d.	n.d.	n.d.	0.12 ± 0.01 b	n.d.	n.d.	0.12 ± 0.01 b
soybean sprouts	BJ	n.d.	n.d.	n.d.	2.26 ± 0.11	n.d.	n.d.	2.26 ± 0.11 a
	HZ	n.d.	n.d.	n.d.	1.29 ± 0.09	n.d.	n.d.	1.29 ± 0.09 b
	GZ	n.d.	n.d.	n.d.	1.07 ± 0.04	n.d.	n.d.	1.07 ± 0.04 c
daylily	BJ	8.11 ± 0.44 b	103.10 ± 5.21 a	5.11 ± 0.27 a	12.85 ± 1.07 a	8.56 ± 0.45 a	5.55 ± 0.29 b	143.28 ± 3.51 a
	HZ	9.84 ± 0.57 a	84.04 ± 7.22 c	4.03 ± 0.33 b	11.89 ± 0.98 ab	8.33 ± 0.36 a	6.87 ± 0.41 a	125.00 ± 4.22 b
	GZ	7.98 ± 0.35 b	98.86 ± 3.21 b	4.44 ± 0.56 ab	10.64 ± 0.79 b	5.22 ± 0.83 b	4.90 ± 0.28 c	132.04 ± 2.84 b

n.d.: not detected (below LOD); tr: trace (below LOQ); BJ: Beijing; HZ: Hangzhou; GZ: Guangzhou. Values (mean ± SD, *n =* 3) of the same compound were showed in the table. Values followed by a different letter (a, b, c) represent a statistically significant difference in the same vegetable amongst the three regions (*p* < 0.05).

**Table 2:** Determination of conjugated phenolic acid content in seven commonly consumed vegetables in China by UPLC-MS/MS (mg/kg FW).

Vegetables	Region	Caffeic Acid	Ferulic Acid	*p*-Coumaric Acid	Sinapic Acid	Gallic Acid	*p*-Hydroxybenzoic Acid	Protocatechuic Acid	Syringic Acid	2,3,4-Trihydroxybenzoic Acid	Vanillic Acid	Total Conjugated Phenolic Acid
kidney bean	BJ	1.42 ± 0.05 a	1.17 ± 0.05 c	1.17 ± 0.32 c	0.32 ± 0.01 b	2.35 ± 0.08 c	n.d.	n.d.	n.d.	0.54 ± 0.04 a	n.d.	6.97 ± 0.37 b
	HZ	0.79 ± 0.03 b	2.69 ± 0.08 b	4.99 ± 0.70 b	1.42 ± 0.06 a	3.77 ± 0.17 a	n.d.	n.d.	n.d.	0.54 ± 0.03 a	n.d.	14.20 ± 0.72 a
	GZ	0.64 ± 0.02 c	3.00 ± 0.12 a	6.70 ± 0.05 a	0.29 ± 0.02 b	2.59 ± 0.14 b	n.d.	n.d.	n.d.	0.56 ± 0.02 a	n.d.	13.78 ± 0.55 a
cow pea	BJ	0.97 ± 0.04 c	4.55 ± 0.21 c	119.69 ± 3.47 c	0.26 ± 0.02 b	0.78 ± 0.02 a	n.d.	1.71 ± 0.05 c	n.d.	n.d.	n.d.	127.96 ± 4.27 c
	HZ	1.4 ± 0.03 a	8.93 ± 0.37 a	134.31 ± 2.99 a	0.31 ± 0.02 a	0.43 ± 0.02 c	n.d.	3.25 ± 0.16 a	n.d.	n.d.	n.d.	148.63 ± 3.44 a
	GZ	1.28 ± 0.02 b	7.28 ± 0.28 b	124.49 ± 4.22 b	0.28 ± 0.02 ab	0.62 ± 0.03 b	n.d.	2.16 ± 0.16 b	n.d.	n.d.	n.d.	136.11 ± 3.68 b
snow pea	BJ	n.d.	2.60 ± 0.12 b	44.36 ± 1.77 a	17.82 ± 0.60 a	0.28 ± 0.01 b	n.d.	2.51 ± 0.12 a	n.d.	n.d.	n.d.	67.57 ± 2.33 a
	HZ	n.d.	3.23 ± 0.13 a	38.84 ± 2.05 b	14.17 ± 0.13 b	0.34 ± 0.03 a	n.d.	2.41 ± 0.03 a	n.d.	n.d.	n.d.	58.99 ± 2.07 b
	GZ	n.d.	2.22 ± 0.07 c	35.85 ± 2.62 b	10.01 ± 0.46 c	0.32 ± 0.02 a	n.d.	1.83 ± 0.05 b	n.d.	n.d.	n.d.	50.23 ± 2.78 c
hyacinth bean	BJ	0.20 ± 0.00 a	2.12 ± 0.05 a	1.10 ± 0.04 a	n.d.	0.09 ± 0.03 b	n.d.	1.30 ± 0.04 b	n.d.	n.d.	n.d.	4.81 ± 0.12 a
	HZ	0.12 ± 0.01 b	0.50 ± 0.01 c	0.72 ± 0.03 c	n.d.	0.06 ± 0.00 b	n.d.	1.36 ± 0.12 ab	n.d.	n.d.	n.d.	2.76 ± 0.11 b
	GZ	0.12 ± 0.04 b	1.30 ± 0.44 b	0.94 ± 1.24 ac	n.d.	0.17 ± 0.01 a	n.d.	1.57 ± 0.18 a	n.d.	n.d.	n.d.	4.10 ± 1.05 a
green soy bean	BJ	n.d.	46.61 ± 2.09 a	2.82 ± 0.13 c	42.01 ± 0.79 a	0.48 ± 0.02 a	1.82 ± 0.05 a	1.00 ± 0.07 b	102.46 ± 3.58 a	n.d.	41.59 ± 0.79 a	238.79 ± 6.88 a
	HZ	n.d.	39.92 ± 1.28 b	5.74 ± 0.13 a	32.93 ± 1.27 c	0.46 ± 0.02 a	1.01 ± 0.03 b	1.14 ± 0.03 a	87.45 ± 3.15 b	n.d.	32.82 ± 1.26 b	201.47 ± 5.33 b
	GZ	n.d.	32.67 ± 1.79 c	4.15 ± 0.18 b	36.99 ± 2.14 b	0.37 ± 0.02 b	0.76 ± 0.10 c	0.92 ± 0.03 b	89.84 ± 3.24 b	n.d.	26.88 ± 1.07 c	192.58 ± 5.22 b
soybean sprouts	BJ	0.49 ± 0.02 b	14.58 ± 0.66 a	18.38 ± 1.04 a	2.98 ± 0.30 b	0.38 ± 0.01 b	2.18 ± 0.10 b	1.03 ± 0.53 ab	n.d.	0.60 ± 0.10 b	10.32 ± 0.52 a	50.94 ± 1.99 a
	HZ	0.57 ± 0.02 a	7.22 ± 0.27 c	15.10 ± 0.67 b	2.33 ± 0.22 c	0.53 ± 0.02 a	1.84 ± 0.08 c	1.38 ± 0.02 a	n.d.	0.87 ± 0.03 a	8.53 ± 0.29 b	38.37 ± 2.09 c
	GZ	0.43 ± 0.01 c	8.08 ± 0.32 b	15.98 ± 0.72 b	3.45 ± 0.12 a	0.26 ± 0.01 c	2.42 ± 0.10 a	1.09 ± 0.04 b	n.d.	0.69 ± 0.02 b	11.56 ± 0.76 a	43.96 ± 1.87 b
daylily	BJ	69.54 ± 2.42 a	40.10 ± 1.54 a	146.31 ± 4.22 a	0.92 ± 0.04 a	2.16 ± 0.07 b	17.39 ± 0.71 b	22.40 ± 0.78 a	0.54 ± 0.01 b	1.38 ± 0.06 ab	12.89 ± 1.24 a	313.63 ± 5.61 a
	HZ	73.42 ± 2.85 a	37.86 ± 1.24 a	134.66 ± 2.79 b	0.81 ± 0.02 b	2.68 ± 0.11 a	14.92 ± 0.44 c	22.27 ± 1.28 a	0.67 ± 0.02 a	1.53 ± 0.10 a	11.76 ± 0.55 a	300.58 ± 4.21 b
	GZ	70.7 ± 2.69 a	39.62 ± 1.56 a	149.61 ± 5.21 a	1.02 ± 0.38 ab	1.77 ± 0.11 c	20.32 ± 1.25 a	14.94 ± 0.95 b	0.55 ± 0.10 b	1.30 ± 0.04 b	8.82 ± 0.72 b	308.65 ± 5.92 ab

n.d.: not detected (below LOD); BJ: Beijing; HZ: Hangzhou; GZ: Guangzhou. Values (mean ± SD, *n =* 3) of the same compound were showed. Values followed by a different letter (a, b, c) represent a statistically significant difference in the same vegetable amongst the three regions (*p* < 0.05).

**Table 3:** Determination of bound phenolic acid content in seven commonly consumed vegetables in China by UPLC-MS/MS (mg/kg FW).

Vegetables	Region	Caffeic Acid	Ferulic Acid	*p*-Coumaric Acid	Sinapic Acid	*p*-Hydroxybenzoic Acid	Protocatechuic Acid	Syringic Acid	Vanillic Acid	Total Bound Phenolic Acid
kidney bean	BJ	n.d.	n.d.	0.54 ± 0.03 a	n.d.	n.d.	n.d.	n.d.	n.d.	0.54 ± 0.03 a
	HZ	n.d.	n.d.	0.59 ± 0.08 a	n.d.	n.d.	n.d.	n.d.	n.d.	0.59 ± 0.08 a
	GZ	n.d.	n.d.	0.42 ± 0.09 a	n.d.	n.d.	n.d.	n.d.	n.d.	0.42 ± 0.09 a
cow pea	BJ	n.d.	n.d.	9.55 ± 0.06 a	n.d.	n.d.	n.d.	n.d.	n.d.	9.55 ± 0.06 a
	HZ	n.d.	n.d.	7.59 ± 0.31 b	n.d.	n.d.	n.d.	n.d.	n.d.	7.59 ± 0.31 b
	GZ	n.d.	n.d.	4.41 ± 0.18 c	n.d.	n.d.	n.d.	n.d.	n.d.	4.41 ± 0.18 c
snow pea	BJ	n.d.	0.14 ± 0.01 a	1.48 ± 0.03 a	0.29 ± 0.01 a	n.d.	4.35 ± 0.25 b	n.d.	n.d.	6.26 ± 0.28 b
	HZ	n.d.	0.14 ± 0.01 a	1.52 ± 0.07 a	0.28 ± 0.01 a	n.d.	5.30 ± 0.10 a	n.d.	n.d.	7.20 ± 0.14 a
	GZ	n.d.	0.08 ± 0.01 b	0.56 ± 0.01 b	0.23 ± 0.02 b	n.d.	2.86 ± 0.11 c	n.d.	n.d.	3.73 ± 0.12 c
hyacinth bean	BJ	1.74 ± 0.05 b	0.23 ± 0.01 a	0.24 ± 0.01 b	n.d.	n.d.	0.11 ± 0.01 a	n.d.	n.d.	2.32 ± 0.07 b
	HZ	1.34 ± 0.04 c	0.21 ± 0.01 a	0.28 ± 0.01 a	n.d.	n.d.	0.12 ± 0.01 a	n.d.	n.d.	1.95 ± 0.07 c
	GZ	4.41 ± 0.23 a	0.10 ± 0.00 b	0.18 ± 0.01 c	n.d.	n.d.	0.08 ± 0.01 b	n.d.	n.d.	4.77 ± 0.25 a
green soy bean	BJ	n.d.	22.09 ± 1.32 a	3.77 ± 0.17 b	16.34 ± 0.69 a	0.78 ± 0.05 a	n.d.	45.10 ± 2.18 a	20.67 ± 1.33 a	108.75 ± 3.57 a
	HZ	n.d.	18.33 ± 0.89 b	1.32 ± 0.56 c	13.54 ± 0.52 b	0.80 ± 0.03 a	n.d.	33.40 ± 1.97 c	18.17 ± 0.73 b	85.56 ± 2.88 b
	GZ	n.d.	12.91 ± 0.61 c	7.81 ± 0.37 a	12.85 ± 0.49 b	0.81 ± 0.03 a	n.d.	36.87 ± 1.24 b	12.65 ± 0.45 c	83.9 ± 2.54 b
soybean sprouts	BJ	n.d.	0.84 ± 0.03 b	1.22 ± 0.05 a	n.d.	0.42 ± 0.01 a	n.d.	17.63 ± 0.76 a	4.68 ± 0.16 a	24.79 ± 1.55 a
	HZ	n.d.	0.71 ± 0.04 c	0.84 ± 0.03 b	n.d.	0.44 ± 0.02 a	n.d.	10.22 ± 0.47 c	3.51 ± 0.12 b	15.72 ± 0.77 c
	GZ	n.d.	1.13 ± 0.06 a	0.64 ± 0.02 c	n.d.	0.44 ± 0.01 a	n.d.	14.98 ± 0.39 b	3.41 ± 0.15 b	20.60 ± 1.02 b
daylily	BJ	7.98 ± 0.36 a	8.23 ± 0.37 a	22.43 ± 1.04 a	n.d.	2.00 ± 0.05 b	3.02 ± 0.03 a	0.07 ± 0.01 a	2.31 ± 0.10 a	46.04 ± 2.01 a
	HZ	5.88 ± 0.30 b	5.24 ± 0.39 b	20.15 ± 1.12 b	n.d.	2.92 ± 0.14 a	2.51 ± 0.04 b	0.05 ± 0.01 ab	1.87 ± 0.06 b	38.62 ± 1.28 b
	GZ	5.18 ± 0.31 c	4.71 ± 0.22 b	16.31 ± 0.77 c	n.d.	1.38 ± 0.04 c	2.38 ± 0.06 c	0.04 ± 0.01 b	1.55 ± 0.05 c	31.55 ± 1.27 c

n.d.: not detected (below LOD); BJ: Beijing; HZ: Hangzhou; GZ: Guangzhou. Values (mean ± SD, *n =* 3) of the same compound were showed. Values followed by a different letter (a, b, c) represent a statistically significant difference of the same vegetable amongst the three regions (*p* < 0.05).

## Supplementary Materials

The following are available online, Figure S1. Loading biplot of seven vegetables and phenolic acids. Table S1. The multiple reactions monitoring (MRM) parameters for phenolic acids.
