# Supplementary material for: Characterization of Free, Conjugated, and Bound Phenolic Acids in Seven Commonly Consumed Vegetables
Source: Molecules. 2017 Nov 1;22(11):1878. doi: 10.3390/molecules22111878 (PMC6150285; doi:10.3390/molecules22111878)
Supplement: Supplementary file 1 [file molecules-22-01878-s001.zip › molecules-231399-supplementary-proofreading/Table S1.docx]

Table S1. The multiple reactions monitoring (MRM) parameters for phenolic acids

| **Phenolic compounds** | **Retention Time (min)** | **Parent ion (*m/z*)** | **Daughter ion (*m/z*)** | **Cone voltage(V)** | **Collision energy(V)** |
| --- | --- | --- | --- | --- | --- |
| Gallic acid | 2.64 | 169 | 79*, 125 | 30 | 25/16 |
| 3,5-Dihydroxybenzoic acid | 3.47 | 153 | 109*, 133 | 40 | 12/10 |
| Neochlorogenic acid | 3.60 | 353 | 191*, 179 | 32 | 20/18 |
| Protocatechuic acid | 3.62 | 153 | 109*, 133 | 52 | 12/8 |
| 2,3,4-Trihydroxybenzoic acid | 3.72 | 169 | 151*, 125 | 20 | 14/12 |
| Chlorogenic acid | 4.20 | 353 | 191*, 179 | 30 | 18/15 |
| *p*-Hydroxybenzoic acid | 4.49 | 137 | 93*, 94 | 38 | 14/10 |
| Gentisic acid | 4.57 | 153 | 109*, 133 | 34 | 14/9 |
| Caffeic acid | 4.77 | 179 | 135*, 117 | 27 | 20/15 |
| Vanillic acid | 4.88 | 167 | 152*, 123 | 22 | 10/15 |
| Syringic acid | 4.99 | 197 | 182*, 167 | 20 | 18/14 |
| Pyrocatechuic acid | 5.09 | 153 | 109*, 133 | 40 | 12/10 |
| Ellagic acid | 5.63 | 301 | 229*, 145 | 82 | 32/26 |
| *p*-coumaric acid | 5.66 | 163 | 119*, 93 | 30 | 25/16 |
| Sinapic acid | 6.00 | 223 | 208*, 164 | 30 | 16/14 |
| Ferulic acid | 6.02 | 193 | 134*, 178 | 32 | 18/13 |
| Isoferulic acid | 6.20 | 193 | 178*, 134 | 20 | 16/12 |
| *m*-Coumaric acid | 6.20 | 163 | 119*, 93 | 38 | 24/14 |
| *o*-Coumaric acid | 6.71 | 163 | 119*,93 | 20 | 30/12 |
| Salicylic acid | 7.09 | 137 | 93*, 94 | 42 | 14/10 |
| Cinnamic acid | 7.68 | 149 | 131*,103 | 28 | 20/12 |

*Used for quantitation
