# Supplementary figures and images for: Characterization of Free, Conjugated, and Bound Phenolic Acids in Seven Commonly Consumed Vegetables
Source: Molecules. 2017 Nov 1;22(11):1878. doi: 10.3390/molecules22111878 (PMC6150285; doi:10.3390/molecules22111878)

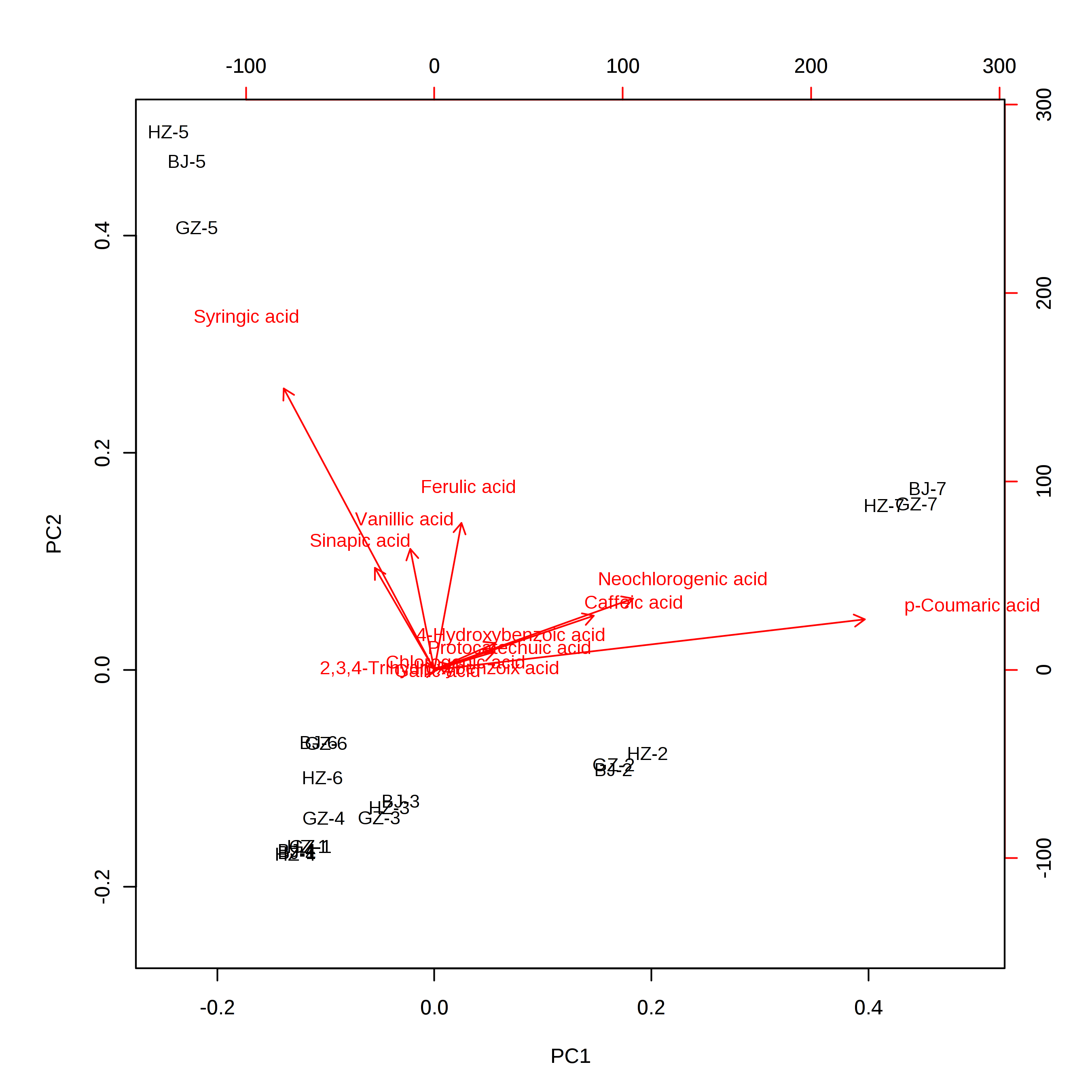

Supplement: Supplementary file 1 [file molecules-22-01878-s001.zip › molecules-231399-supplementary-proofreading/Figure S1.tiff]
